# Puerperal Septicæmia

**Published:** 1893-06

**Authors:** J. I. Darby

**Affiliations:** Americus, Ga.


ATLANTA
MEDICAL AND SURGICAL JOURNAL.

VOL. X. JUNE, 1893. No. 4.

EDITED BY
LUTHER B. GRANDY, M. D., AND MILLER B. HUTCHINS, M. D.,
WITH THE CO-OPERATION OF
H. V. M. MILLER, M. D., LL. D., VIRGIL O. HARDON, M. D.,
FLOYD W. McRAE, M. D., AND ARTHUR G. HOBBS, M. D.

### *ORIGINAL COMMUNICATIONS.*

### PUERPERAL SEPTICÆMIA.*

BY J. I. DARBY, M. D.,
AMERICUS, GA.

Puerperal septicæmia has in all civilized countries been the bane of parturient woman and opened a broad gateway into eternity until within very recent years. We have no very satisfactory means of ascertaining the time when this fearful disease was first recognized; but basing our opinions of it upon the present knowledge of its etiological factors, we are justified in supposing that it was necessarily a very common one in the days of man's primitive conditions, as his environments at that early time were such as to make quite common this horrible malady. While it is to some extent properly understood and intelligently treated in many portions of civilized countries, it is and will continue to be the scourge of lying-in woman in the more benighted portions of earth. Various and conflicting theories have, from its earliest recognition, existed

*Read at the Georgia Medical Association, April 19, 1893.

concerning its cause. Many pages have been written to prove its contagious character, and much has been said concerning its treatment, but until within the two last decades the medical profession possessed but meager knowledge concerning its true cause and proper treatment. And even now, there is apparent want of unity upon the subject; however, I think, it has been pretty satisfactorily established that the same causes which produce septic conditions in the male subject will, under similar conditions, produce the same result in the female, and that puerperal septicæmia only differs from surgical septicæmia in that the seat of lesion in the former is through the parturient canal, whereas in the latter it may be through lesions on any portion of the body. In a word, septicæmia in the parturient female results from the introduction into the system of the same pyogenic microbes which produce septic conditions in the male, or non-puerperal female. Microscopists, in their examinations of the blood and tissues of those sick with the disease, find the streptococcus and staphylococcus pyogenes coexisting, but it is known that the streptococcus or chain-like bacilli are the etiological factors and that the staphylococcus exists only as a secondary product, brought about by the streptococcus preparing the parts for their growth and proliferation. Repeated inoculations with the pus and ptomaines from septic wounds into animals produce uniform and characteristic results. Ptomaines from an ordinary traumatism will, when introduced into the abraded genital tract in sufficient quantity within the first five days after parturition, produce puerperal septicæmia. Now I am not by any means ignorant of the fact that quite a goodly number of our very respectable brethren believe in two separate and distinct varieties of puerperal septicæmia. One variety produced by the pyogenic microbes, of which I have just spoken, and the other variety caused by a septic toxæmia due to the effects of chemical poisons evolved during the putrefactive decomposition of certain organic substances, especially of nitrogenous animal products. But with all the scientific light of the closing decade of the nineteenth century shining down upon the subject and the magnifying power of the modern microscope contributing its aid, we are unable to discover any etiological factors other than the pyogenic microbe.

We do not believe it possible for organic animal substances to undergo decomposition until they are brought into contact with putrescent bacilli. We believe that sufficient experiments have been made upon the living human body to prove beyond all doubt that portions of the body may be entirely deprived of nerve and blood supply and yet not undergo any degree of decomposition or putrefaction as long as there is no channel open through which bacteria can find their way to the parts. Therefore we can accept but one variety of septicæmia, be it of *puerperal* or *surgical* origin.

In the puerperal woman the genital tract is abraded and made a suitable field for the rapid production and absorption of septic germs, which, finding their way in sufficient numbers through the lymphatics into the blood and tissues, produce their characteristic manifestations in decided pathological conditions. But in order that she may suffer from these micro-organisms, it is necessary for them to come in contact with wounded surfaces of the genital tract, usually within the first five days after her confinement, for after that time in previously healthy women, resolution has so far taken place as to preclude the possibility, in most instances, of the disease being inaugurated. After this time the wounded surfaces are granulating and not in a condition to absorb pyogenic germs. The primary symptoms of puerperal septicæmia are identically those of septicæmia occurring after traumatisms in the male subject, or the non-puerperal female, and the conditions which manifest themselves subsequently, differing from the ordinary surgical septicæmia, are due to the peculiarity of the organs locally involved.

The disease is usually ushered in with a chill, occurring between second and fifth day after parturition and may be of variable intensity, and the fever following it is peculiar, in that the thermometric curve is irregular throughout its entire course. We seldom witness decided and pronounced intermissions, but frequently notice evening exacerbations, attended with profuse perspirations of acid odor.

Diarrhœa is an early and often persistent symptom, metastatic abscesses sometimes occur, but when they do the condition is one of pyæmia rather than septicæmia. The pulse, which is usually full in the beginning of the attack, soon becomes small and rapid, and, in cases tending towards a fatal issue, small and dicrotic. Thirst is a constant and distressing symptom. The tongue usually presents a red and smooth, shining appearance from the second or third day in bad cases. The temperature immediately following the initial chill generally runs high, frequently reaching 105 degrees Fahrenheit within the first twenty-four hours and maintaining a high grade throughout the entire course, but characterized by morning remissions of slight character. Irritable stomach is nearly always present, and vomiting is often an uncontrollable symptom. The pulse very soon becomes quick and small, and in many instances is decidedly compressible from the beginning of the attack. The cadaveric odor of the body is noticeable in all severe cases, and frequently distresses the patient as well as those who are compelled to come in contact with her. The urine is scanty from the outset of the disease and presents a decided yellow appearance. The chill is frequently repeated several times during the course of the attack. One of the most distressing symptoms in severe cases is the pain experienced in the pelvic and abdominal regions, produced by the local inflammation in these parts. Abscesses do not always occur, especially when the case is treated promptly and intelligently from the beginning. I have seen quite a good many cases where the peritoneum was but slightly, if at all, involved, and yet the symptoms were such as to justify a diagnosis of puerperal septicæmia. The physician, who, by his careful management of his parturient patients, prevents septicæmia, deserves more honor than he who treats it well after it has been inaugurated, for there must necessarily be under all forms and methods of treatment a very large per cent. of fatal cases; in the first half of the present century the mortality attending it was simply frightful. Recognizing puerperal septicæmia, as I do, to be in all cases due to the introduction of a pyogenic microbe into the tissues of a parturient woman, and in no instance due to chemical products independent of these organisms, it can readily be seen that I would expect to do far better services in trying to prevent the disease than in treating it after it has been inaugurated. Indeed, I believe it to be within the power of the physician to limit the number of these cases to an infinitesimal few, if not to stamp them out entirely, by a judicious use of antiseptic remedies.

The hospital reports of Philadelphia, New York and other cities, where antiseptic midwifery is practiced, show that the mortality rate has decreased from an alarming figure to almost nothing, and why is this so? If the disease could be produced by chemical ptomaines, generated in the uterus or other part of the genital apparatus, independent of germs, why is it that in those institutions where the parturient patient is protected from them she never contracts septicæmia of any variety? I do not believe it possible for toxic ptomaines to generate in the system independent of microbes, and in the instances where such has been supposed to be the case I believe mistakes have been made.

To prevent the patient having septicæmia she should be properly prepared for her confinement by being well bathed and placed in a clean gown and bed at the beginning of labor. The bowels should be well evacuated by enema, and the vulva and other external parts well washed with good soap and warm water and afterwards rinsed well with a solution of bichloride mercury, 1 to 2000. This can readily be done by the accoucheur supplying the nurse with a few $7\frac{1}{2}$ gr. tablets of mercury, which he should always carry in his obstetric satchel. The tablet dissolved in a quart of warm water makes the desired solution. After this preparation of the external parts by the nurse, the physician should give a vaginal douche of the antiseptic solution, after which he should, with thoroughly cleansed hands, proceed to make a digital examination and if possible ascertain the presentation, after which there should be no further introduction of the finger until the rupture of the membranes or the character of the pain indicates the necessity for so doing. Sometimes, however, when the first stage of labor is tedious and prolonged beyond the ordinary period of time, it is justifiable and even necessary to acquaint ourselves with the condition of the parts by repeated digital examinations, but they should in every instance be preceded by a careful washing of the hands, and in very protracted cases be followed by an antiseptic vaginal douche. When the second stage of labor has been completed the placenta should usually be removed within the next twenty-five minutes, by Crede's method if possible; if not possible by this method, it should be done in such manner as the nature of the complication will require, taking great care to inflict as little violence upon the soft parts as the nature of the case will allow. After the placenta has been removed and the uterus well contracted, we next irrigate the vagina again with our same antiseptic solution and apply a piece of bichloride gauze over the vulva, which should be replaced several times during each day with other pieces of the same material. When instruments have to be used they should be carefully prepared and not allowed to come in contact with the bedding or other septic material after being dipped into antiseptic solutions, and if they are anointed at all it should be with sterilized vaseline or other lubricant properly treated with carbolic acid or bichloride mercury. In either natural or instrumental labors the accoucheur should not fail to carefully examine the external organs of the patient, and if they are lacerated or seriously abraded he should treat them according to indications. If the perineum is lacerated he should repair it at once; if the vulva is contused or injured it should be cleansed and treated with iodoform collodion. The antiseptic vaginal douches should be kept up by the nurse for at least five days after delivery, and might with propriety be used daily throughout the entire lying-in month. The observance of the above precautions will usually be sufficient to insure the woman against septic infection, and they can be carried out in private practice.

I know that thousands of women have been delivered of their offspring without any of the above details being carried out, and made good recoveries, and such will continue to be the case; but I further know, that there is not a practitioner of much experience who has not lost cases of septicæmia which could have been prevented by proper antiseptic treatment. I admit that many women who never heard of antiseptic midwifery have done quite as well as those attended by the greatest disciple of antisepticism, but it is the few who are lost for the want of the proper use of these precautionary measures we are seeking to save. We have nothing to lose by our prophylaxis and much to gain, and if we only save one human life during our whole medical career and do not injure any, we have made ourselves benefactors to our race.

When from any reason the disease has been inaugurated, we must proceed to treat it promptly, recognizing that we have one of the most dangerous maladies of which human flesh is heir to combat. *The treatment* consists in the judicious use of antiseptics and such other remedies as will properly sustain the patient's vitality. High temperature, being one of the dangerous phenomena, should be combated with phenacetine and sulphate of quinine, or what may in many cases be better, salicylate of soda or salol in ten or fifteen grain doses every three hours, or by the application of cold water frequently applied. But one of the most important measures to be resorted to in the beginning of the treatment of cases due to retained secundines, is the thorough and effectual removal of all foreign substances from the vagina and uterine cavity, and this can only be properly done by making the vagina aseptic, by the liberal use of bichloride solution, and then passing the dull curette into the cavity and carefully scraping its walls, so as to remove all exfoliated endometrium and other *débris*, which may be undergoing putrefactive decomposition. After carefully scraping the cavity with the curette, the glass nozzle of a clean fountain syringe should be introduced into it and the part thoroughly irrigated with a hot 1 to 3000 bichloride mercury solution, taking especial care to see that the os is sufficiently dilated to allow a free and easy return of the fluid from the uterine cavity; after which the uterus and vagina should be filled with iodoform gauze, or the uterine cavity may have an iodoform suppository introduced into it, and the vagina lightly packed with the gauze, and let remain for a period of about twelve hours, after which the irrigating treatment should be gone through with at least twice in every twenty-four hours, leaving off the curetting usually after the first time, provided it was well done in the beginning. But if there is no evidence of retained secundines the curetting should be omitted entirely.

All abrasions should receive careful cleansing and iodoform applications. Abscesses should be carefully opened, washed out and dressed with iodoform gauze and drainage tubes, in suitable cases.

The compressible pulse indicates impending danger from heart failure, and strongly suggests the use of digitalis and other cardiac stimulants, which should be administered freely from the beginning. Whisky and brandy should be given without stint, regarding their effects rather than the quantity. The pain, which is often a distressing symptom, should be relieved by hypodermics of morphine and atropine, and the bowels kept gently lax with saline cathartics if necessary, but we are generally troubled more by persistent diarrhœa and have to combat that with acetate of lead, opium and other astringents, with the constant use of turpentine stupes and other hot applications over the pelvic and abdominal regions. In some instances it *might be well* to apply a blister. Whisky and brandy not only stimulate the heart and counteract the toxic ptomaines set free in the system, but act as a food also; we should give it freely combined with milk and eggs as well as in the form of toddies. Beef tea and other meat broths should be given as freely as the condition of the patient's stomach will allow. When the stomach cannot retain food the rectum and colon should be resorted to, but in order to not offend these organs, the quantity injected at each time should be small, consisting of raw egg, milk and whisky in combination. When the more violent symptoms have been controlled and the patient is improving, I usually prescribe dilute hydrochloric acid and quinine. Muriate and tincture of iron in fifteen or twenty drop doses, three times daily, is an excellent remedy for the convalescent patient, when the bowels are in a condition to retain it. Compound syrup of hypophosphites in teaspoonful doses three times daily also benefit many and should be given.

